# Human MettL3–MettL14 complex is a sequence-specific DNA adenine methyltransferase active on single-strand and unpaired DNA in vitro

**DOI:** 10.1038/s41421-019-0136-4

**Published:** 2019-12-24

**Authors:** Clayton B. Woodcock, Dan Yu, Taraneh Hajian, Jia Li, Yun Huang, Nan Dai, Ivan R. Corrêa, Tao Wu, Masoud Vedadi, Xing Zhang, Xiaodong Cheng

**Affiliations:** 1 Department of Epigenetics and Molecular Carcinogenesis, University of Texas MD Anderson Cancer Center, Houston, TX USA; 2 Structural Genomics Consortium, University of Toronto, Toronto, ON Canada; 3 Center for Epigenetics & Disease Prevention, Institute of Biosciences and Technology, Texas A&M University, Houston, TX 77030 USA; 4 New England Biolabs, Inc, Ipswich, MA 01938 USA; 5 Department of Molecular and Human Genetics, Baylor College of Medicine, Houston, TX 77030 USA; 6 Department of Pharmacology and Toxicology, University of Toronto, Toronto, ON M5S 1A8 Canada

**Keywords:** Methylation, Nucleosomes

Dear Editor,

Human MettL3 and MettL14 are two members of the MT-A70 family of S-adenosyl-l-methionine (SAM)-dependent methyltransferases (MTases). They form a heterodimer^1^ known to function as an mRNA adenine-N6 MTase (ref. ^2^ and references therein). In addition, MettL3 and MettL14 associate with chromatin and localize to the transcription start sites of active genes^3^. Here we show human MettL3–MettL14 complex is active in vitro as a DNA adenine-N6 MTase and methylates GGACT in single-strand DNA and double-strand DNA-containing mismatches.

DNA methylation in bacteria and archaea is common, occurring at carbon C5 of cytosine ring or exocyclic amino groups of cytosine (at N4) and adenine (at N6). The bacterial ‘orphan’ MTases—which are not coupled with a restriction endonuclease as part of a self-defense restriction-modification system (Supplementary Ref. S1)—are generally involved in epigenetic gene regulation, chromosome replication and DNA repair. Examples of orphan MTases include the DNA adenine MTase (Dam) in *Escherichia coli* and cell cycle-regulated DNA MTase (CcrM) in *Caulobacter crescentus* which are, respectively, responsible for maintenance of adenine methylation of GATC or GAnTC (n = any nucleotide) immediately after replication (Supplementary Ref. S2, S3).

In mammals, the epigenetic DNA methylation marks have been generated and maintained by DNA cytosine-C5 MTases Dnmt1 and Dnmt3 family (Supplementary Ref. S4), whereas DNA adenine methylation was reported only recently. Low levels of N6-methyladenine (N6mA) in DNA have been observed in mouse embryonic stem cells^4^ and human glioblastoma^5^. However, the observations of existence of DNA N6mA in the genomes of higher organisms are controversial (Supplementary Ref. S4.1-2) and the identity of the mammalian DNA adenine MTase(s) has not yet been convincingly established. Whereas mammalian HemK2 had been documented to be a DNA adenine-N6 MTase (Supplementary Ref. S5) (renamed as N6AMT1), we and others found that human HemK2 is not active on DNA (Supplementary Ref. S6, S7).

The following considerations prompted us to investigate whether human MettL3–MettL14 heterodimer (termed MettL3-14 thereafter) also possesses methyl transfer activity on DNA adenine. First, while MettL3 is preferentially enriched at the 3′ end of protein-coding genes (Supplementary Ref. S8), echoing its involvement in mRNA adenine methylation, MettL3 and MettL14 are also associated with chromatin and localize to the transcriptional start sites of active genes^3^. Reanalyzing published ChIP-seq datasets of MettL3 and MettL14 from human leukemia MOLM13 cells showed that 37% of MettL3 and 85% of MettL14 binding sites contain DNA sequences equivalent to the RNA-recognition motif of MettL3-14, represented by RR**A**CH (R = G/A and H = A/C/U in RNA and A/C/T in DNA) (Supplementary Fig. S1). Upon ultraviolet irradiation, MettL3 and MettL14 are recruited rapidly (within 2 min) to the damaged sites, and MettL3 activity is required for the DNA repair^6^. MettL14 has been reported to recognize trimenthylation of histone lysine 36 (H3K36me3)^7^ and loss of MettL3 results in loss of trimethylation of histone H3 lysine 4 (H3K4me3)^8^. In addition, the *Drosophila MettL3* homolog *Ime4* (*I*nducer of *me*iosis *4*) is localized to the sites of transcription (Supplementary Ref. S9).

Second, while mammalian MettL3-14 is active on single-strand mRNA^2^, many nucleic acids-modifying enzymes are able to modify both DNA and RNA (ref. ^9^ and references therein), including members of AlkB family involved in the direct reversal of alkylation damage to DNA and RNA (Supplementary Ref. S10), and members of Apobec family of cytidine deaminases (Supplementary Ref. S11). Tet2, one of the ten-eleven translocation proteins initially discovered as DNA 5-methylcytosine (5mC) dioxygenases (Supplementary Ref. S12), mediates oxidation of 5mC in mRNA (Supplementary Ref. S13, S14).

Third, MettL3 and MettL14 belong to a functionally diverse MT-A70 family of SAM-dependent MTases (Supplementary Ref. S15). Another family member (murine MettL4) was reported to be responsible for N6-methyladenine deposition in genic elements corresponds with transcriptional silencing^10^ (though the in vitro enzymatic activity of MettL4 was not reported). In addition, a DNA adenine MTase complex in ciliates (single-celled eukaryotes), consisting of two MT-A70 proteins (MTA1 and MTA9), methylates double-stranded DNA^11^. We note that four out of five nucleotides within the consensus motif of MettL3-14 overlap with the recognition sequence of CcrM (GG**A**CT vs. G**A**nTC), an enzyme active on both double-stranded (ds) and single-stranded (ss) DNA (Supplementary Ref. S16). We thus included CcrM as a positive control. Both CcrM and MettL3-14 are β-class MTases^12^.

We designed three short dsDNA oligos (the oligo numbers refer to our laboratory code and letters T and B designate the top and the bottom strand respectively): #4 contains the CcrM recognition sequence GA(g/c)TC, #5 contains the Dam recognition sequence GATC and used as a negative control, and #6 contains GGACT, the DNA equivalent of the RNA-recognition sequence of MettL3-14 and an overlapping CcrM recognition sequence (GGAcTC) (Fig. 1a). Using purified recombinant enzymes (Supplementary Fig. S2a), under the saturating conditions of high enzyme concentration ([E] = 2 μM) where CcrM completed reactions on its known substrates (oligos #4 and #6), we observed no activities of MettL3-14 on the double-stranded oligos examined (Fig. 1b). However, we did observe strong activity of MettL3-14 on single-stranded 6 T (containing GGACT), much reduced activities on 4 T (containing GGAGT) and 4B (AGACT), and no activity on oligos having two substitutions within the recognition, e.g. 6B (AGAGT) or 5 T and 5B (GGATC) (Fig. 1c, d). Furthermore, we confirmed that MettL3-14 has no activity on an oligo-containing G and C only (control 1), and no activity on A-containing oligos without matching consensus sequence (controls 2 and 3) (Supplementary Fig. S2b). Importantly, single A-to-G substitution in oligo 6 T abolished the activity (Supplementary Fig. S2c), clearly demonstrating the A within the GGACT recognition sequence is the site of methylation by MettL3-14 complex.

**Fig. 1:**
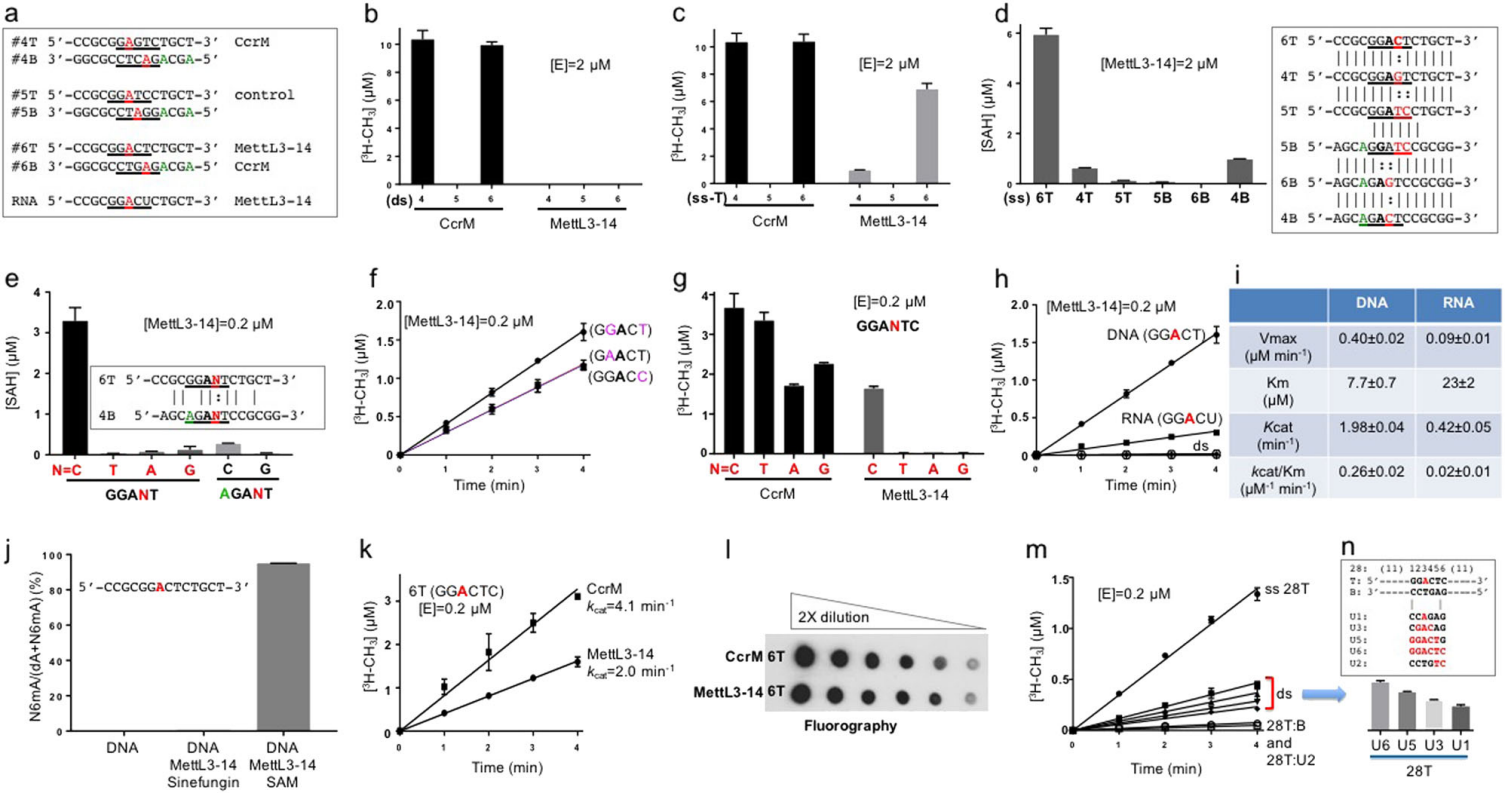
Human MettL3-14 complex is active on ssDNA and mismatched DNA adenine. **a** Olignonuceotides (14-mer) used as substrates. **b** MettL3-14 is not active on dsDNA. The enzyme concentration [E] is noted in each panel. **c**, **d** MettL3-14 is active on ssDNA by two independent assays: incorporation of tritium from ^3^H-SAM into DNA substrate (panel **c**) and formation of byproduct SAH in a bioluminescence assay (panel **d**). Inserted box listed ssDNA sequence alignment. **e** Substitution of conserved cytosine within GGACT diminished Mettl3-14 activity. **f** Replacement of the second guanine-to-adenine (G**A**ACT, red) or the last thymine-to-cytosine (GGAC**C**, blue) retained comparable activity. The two lines fell on top to each other, giving rise to purple. **g** Comparison of CcrM (GAnTC) and MettL3-14 (GGACT) on ssDNA oligo 6 T and its derivatives. **h** Comparison of MettL3-14 on oligo 6 T and its corresponding RNA. **i** Comparison of the kinetic parameters on ssDNA and ssRNA, derived from Supplementary Fig. S2d. **j** Quantitative measurement of N6mA by mass spectrometry, derived from Supplementary Fig. 3. **k**, **l** Comparison of CcrM and MettL3-14 on oligo 6 T. **m**, **n** MettL3-14 is active on 28-bp dsDNA-containing mismatched pairs. Data represent the mean ± SD of three independent determinations (*N* = 3) performed in duplicate.

We tested the sequence specificity for the conserved cytosine after the target adenine (AC) by substituting the C to the other three nucleotides (T, A and G) in the context of oligo 6 T (GGAnT). We used lower enzyme concentration ([E] = 0.2 μM) and shorter reaction time to perform the assays in the linear range. We found that MettL3-14 has no activity on the C-to-T or C-to-A substitutions, and residual activity on the C-to-G substitution (GGAGT) (Fig. 1e). The outer guanine-to-adenine substitution (AGACT) had minor activity, while combining both replacements resulted in total loss of activity (AGAGT) (Fig. 1e). However, replacement of the second guanine-to-adenine (G**A**ACT) or the last thymine-to-cytosine (GGAC**C**) retained comparable activity (Fig. 1f). The preferred sequence (G-g/a-AC-t/c) is consistent with the consensus RNA sequence (RRACH)^2^, particularly the requirement of a cytosine following the target adenine (Supplementary Fig. S1c). As noted above, the oligo 6 T also contains an overlapping CcrM consensus sequence with the cytosine after target adenine corresponds to the variable position (GAnTC). As expected, CcrM methylates oligo 6 T and its derivatives with the N variations (Fig. 1g).

More strikingly, MettL3-14 acts on ssDNA (6 T) faster than that on the corresponding ssRNA under these conditions; but the heterocomplex is inactive on dsDNA or RNA/DNA hybrid (Fig. 1h). Under stead-state kinetics conditions, MettL3-14 methylates the 6 T ssDNA with *k*_cat_ = 1.98 min^−1^ and K_m_ = 7.7 µM (Fig. 1i and Supplementary Fig. S2d). On ssRNA of the same sequence and length, the MettL3-14 methylation rate is ~5-fold slower (*k*_cat_ = 0.42 min^−1^) with ~3-fold weaker affinity for the ssRNA substrate (K_m_ = 23 µM). In other words, MettL3-14 shows 13-fold weaker catalytic efficiency of methylation on an RNA substrate (comparing *k*_cat_/K_m_ value of 0.02 µM min^−1^ for RNA and 0.26 µM min^−1^ for DNA). In addition, we applied quantitative mass spectrometry to monitor the product formation using the single-A-containing ssDNA oligo (6 T) as substrate. We detected ~95% conversion of A-to-N6mA in the presence of SAM, but not for the DNA alone or with Sinefungin (an analog of SAM and a pan-inhibitor against SAM-dependent MTases) (Fig. 1j and Supplementary Fig. S3). Furthermore, the in vitro activity of MettL3-14 on ssDNA is comparable (~2× slower) to that of the well-characterized CcrM on the same substrate (Fig. 1k, l). We note that the preference of MettL3-14 for ssDNA over ssRNA is analogous to that of *E. coli* AlkB, which removes methyl lesions in ssDNA more efficiently than RNA in vitro (Supplementary Ref. S17).

While DNA sequences are base paired in canonical double helix, transient local unwinding of dsDNA does occur during processes of transcription, replication, recombination and DNA repair, such as a transcriptional bubble (Supplementary Ref. S18). The feature of ssDNA in *E. coli* could be induced by stress-induced DNA duplex destabilization (SIDD) enriched in promoter regions^13^ and ssDNA is too a common feature of mammalian genome potentially involved in gene regulation^14^. In *Caulobacter crescentus*, CcrM methylates the adenine of hemimethylated GAnTC sites following replication (Supplementary Ref. S19). Recently we show that CcrM binds DNA by strand-separation of dsDNA and creates a bubble at its recognition site^15^ (Supplementary Fig. S4a), in agreement with CcrM being active on both dsDNA and ssDNA in vitro (Supplementary Ref. S16). In addition, CcrM can accommodate mismatch within or immediate outside of the recognition sequence (Supplementary Ref. S20).

As noted above, MettL3-14 is rapidly recruited to the UV-induced DNA damage sites^6^. We thus asked whether MettL3-14 also has the capacity to methylate dsDNA with mismatches or unpaired region within the recognition sequence. We synthesized a longer DNA molecule (28-bp) with one-to-six unpaired bases flanked by at least eleven base pairs on either side to assure the formation of one complete helical turn of dsDNA (Supplementary Fig. S4b). We first validated that MettL3-14 complex methylates the 28-nt and 14-nt ssDNA about equally (Supplementary Fig. S4c), and that it is completely inactive on the fully paired duplex (as expected). However, partial activity was observed on the 28-bp dsDNA-containing unpaired bases, either 1, 3, 5 or 6 mismatched bases (U1 to U6) centered on the target adenine, while no activity was detected when the mismatched pairs do not include the target A (U2) (Fig. 1m). Interestingly, the level of activity on dsDNA correlates positively with the number of mismatched bases (Fig. 1n) and is not affected by the excess of bottom strand which contains no recognition sequence—added to assure that the top, target strand is fully annealed (Supplementary Fig. S4d).

In summary, we characterized for the first time the in vitro enzymatic activity of mammalian MettL3-14 as a sequence-specific DNA adenine–N6 MTase complex. The complex specifically methylates single-strand DNA and unpaired region (with reduced activity) in the context of double-strand DNA. Additional study will be required to address whether MettL3-14 mediates DNA adenine methylation in vivo and its impact on chromatin organization. Finally, there are ancillary factors, such as Wilms tumor suppressor-associated protein (WTAP), that ultimately determine the cellular functions of MettL3-14. WTAP is required for MettL3-14 localization (Supplementary Ref. S21) and in vivo methylation activity on mRNA (Supplementary Ref. S21.1-3). In addition, WTAP plays a role in both transcriptional (perhaps acting on DNA) and post-transcriptional (perhaps acting on mRNA) regulation of certain cellular genes (Supplementary Ref. S22). How WTAP affects the activity of MettL3-14 on RNA vs DNA requires further study. We note that MettL3 and MettL14 (but not WTAP) are recruited to the damaged sites upon ultraviolet irradiation^6^.

A potential correlation might exist between markedly upregulated N6mA levels in glioblastoma^5^ and stress-induced DNA duplex destabilization^14^. The DNA strand-separation event might facilitate the access to the target base by the MTase complex studied here (as well as other β-class MT-A70 family members). Furthermore, the methyl group covalently attached to the adenine N6 atom—which is directly involved in Watson–Crick A:T base pairing—might in turn compromise DNA stability locally (Supplementary Ref. S23). The destabilized N6mA-containing region might also facilitate the removal of the methyl group of N6mA by the AlkB family of repair enzymes.

## Supplementary information

Supplementary Information

## Supplementary information

**Supplementary Information** accompanies the paper at (10.1038/s41421-019-0136-4).

## References

[1] Liu J (2014). A METTL3-METTL14 complex mediates mammalian nuclear RNA N6-adenosine methylation. Nat. Chem. Biol..

[2] Balacco DL, Soller M (2019). The m(6)A writer: rise of a machine for growing tasks. Biochemistry.

[3] Barbieri I (2017). Promoter-bound METTL3 maintains myeloid leukaemia by m(6)A-dependent translation control. Nature.

[4] Wu TP (2016). DNA methylation on N(6)-adenine in mammalian embryonic stem cells. Nature.

[5] Xie Q (2018). N(6)-methyladenine DNA modification in glioblastoma. Cell.

[6] Xiang Y (2017). RNA m(6)A methylation regulates the ultraviolet-induced DNA damage response. Nature.

[7] Huang H (2019). Histone H3 trimethylation at lysine 36 guides m(6)A RNA modification co-transcriptionally. Nature.

[8] Kuppers DA (2019). N(6)-methyladenosine mRNA marking promotes selective translation of regulons required for human erythropoiesis. Nat. Commun..

[9] Forterre, P. & Grosjean, H. The interplay between RNA and DNA modifications. In *DNA and RNA Modification Enzymes: Structure, Mechanism, Function and Evolution (edited by H. Grosjean) Landes Bioscience*. 259–274 (2009).

[10] Kweon SM (2019). An adversarial DNA N(6)-methyladenine-sensor network preserves polycomb silencing. Mol. Cell.

[11] Beh LY (2019). Identification of a DNA N6-adenine methyltransferase complex and its impact on chromatin organization. Cell.

[12] Malone T, Blumenthal RM, Cheng X (1995). Structure-guided analysis reveals nine sequence motifs conserved among DNA amino-methyltransferases, and suggests a catalytic mechanism for these enzymes. J. Mol. Biol..

[13] Wang H, Noordewier M, Benham CJ (2004). Stress-induced DNA duplex destabilization (SIDD) in the *E. coli* genome: SIDD sites are closely associated with promoters. Genome Res..

[14] Kouzine F (2017). Permanganate/S1 nuclease footprinting reveals Non-B DNA structures with regulatory potential across a mammalian genome. Cell Syst..

[15] Horton JR (2019). The cell cycle-regulated DNA adenine methyltransferase CcrM opens a bubble at its DNA recognition site. Nat. Commun..

