# Supplementary material for: Human MettL3–MettL14 complex is a sequence-specific DNA adenine methyltransferase active on single-strand and unpaired DNA in vitro
Source: Cell Discov. 2019 Dec 24;5:63. doi: 10.1038/s41421-019-0136-4 (PMC6928010; doi:10.1038/s41421-019-0136-4)
Supplement: Supplementary file 1 — Supplementary Information [file 41421_2019_136_MOESM1_ESM.pdf]

Supplementary Information

Woodcock et al.

Human MettL3-MettL14 complex is a sequence specific DNA adenine methyltransferase active on single-strand and unpaired DNA in vitro

Clayton B. Woodcock[1], Dan Yu[1], Taraneh Hajian[2], Jia Li[3], Yun Huang[3], Nan Dai[4], Ivan R. Corrêa Jr[4], Tao Wu[5], Masoud Vedadi[2,6], Xing Zhang[1,7*], Xiaodong Cheng[1,7*]

[1]Department of Epigenetics and Molecular Carcinogenesis, University of Texas MD Anderson Cancer Center, Houston, TX, USA. [2]Structural Genomics consortium, University of Toronto, Toronto, Ontario, Canada. [3]Center for Epigenetics & Disease Prevention, Institute of Biosciences and Technology, Texas A&M University, Houston, TX 77030, USA. [4]New England Biolabs, Inc., Ipswich, MA 01938, USA. [5]Department of Molecular and Human Genetics, Baylor College of Medicine, Houston, TX 77030, USA. [6]Department of Pharmacology and Toxicology, University of Toronto, Toronto, ON, M5S 1A8, Canada

[7]These authors jointly supervised this work: Xing Zhang, Xiaodong Cheng

* e-mail: xzhang21@mdnderson.org; xcheng5@mdanderson.org

Email addresses of all authors:

CBW (CBWoodcock@mdanderson.org); DY (DYu6@mdanderson.org); TH (taraneh.hajian@utoronto.ca); JL (jli@ibt.tamhsc.edu); YH (yun.huang@ibt.tamhsc.edu); ND (Dain@neb.com); IC (correa@neb.com); TW (Tao.Wu@bcm.edu); MV (m.vedadi@utoronto.ca)

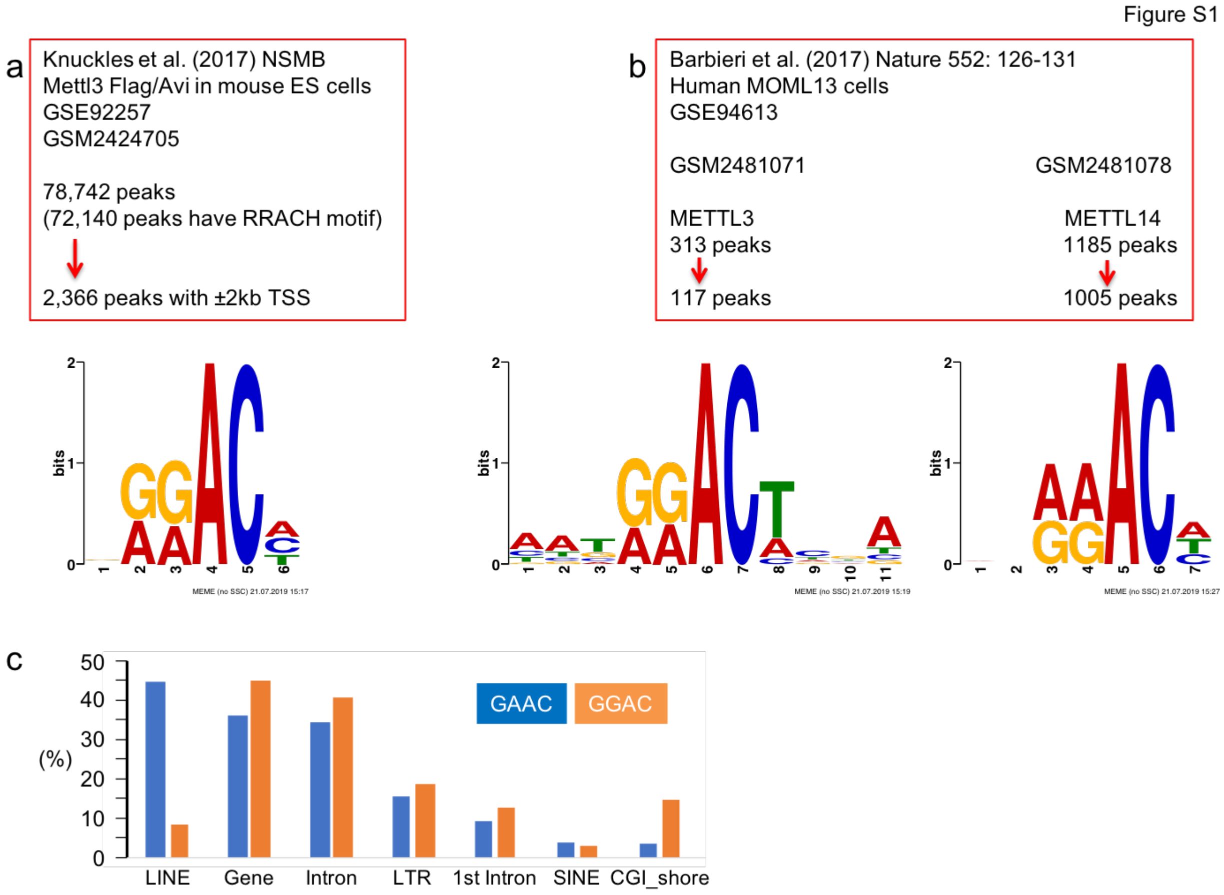

Fig. S1. **a-b**, Motif analysis of MettL3 and MettL14 ChIP-seq peaks with RRACH motif that located within ±2 kb of TSS. The information of MettL3 and MettL14 ChIP-seq analysis in mESC (panel a) and human MOML13 cells (panel b). **c**, The percentage of calculated GGAC or GAAC motif blocks within annotated regulatory elements. The x-axis indicates the regulatory elements, the y-axis represents the percentage of motif blocks in each regulatory elements. An in-house python script (https://github.com/lijiacd985) was used to scan the whole human genome (UCSC hg19 version) to extract the genome coordinated with GGAC or GAAC motif. The motifs that span less than 50 bp were defined as motif blocks. The blocks with length longer than 100 bp were used for further analysis. The distribution of blocks was then analyzed by overlapping with regulatory elements (hg19 version). The numbers of blocks overlapped with every regulatory element were recorded and the percentages of these blocks were used to plot the histogram. LINE: Long interspersed nuclear elements; Gene: coding regions of genes; Intron: intron; LTR, Long terminal repeats present in class I transposable elements; 1st intron, the first intron; SINE, Short interspersed nuclear elements, CGI_shore, CpG island shores.

Figure S2

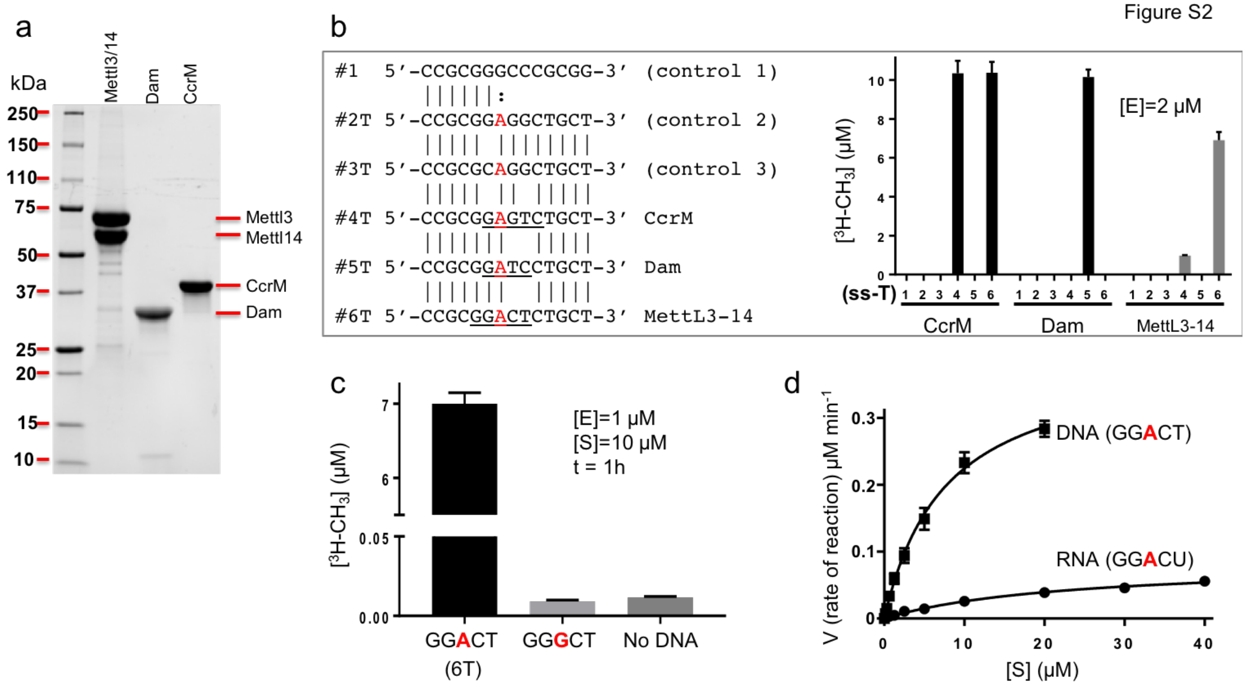

Fig. S2. **a**, Recombinant proteins used in the study. **b**, CcrM, Dam, and Mettl3-14 are not active on control oligos 1, 2 and 3 containing no consensus motifs for CcrM, Dam, or MettL3-14. **c**, MettL3-14 activity is dependent exclusively on target adenine within the consensus sequence. The assays were performed at 37°C and pH 7.5 for 1 h using [E]=1 µM, [S]=10 µM oligos, [SAM]=20 µM. **d**, Comparison of kinetics of MettL3-14 on ssDNA and ssRNA. The assays were performed at 37°C and pH 7.5 for 2 min per reaction using [E]=0.2 µM, [SAM]=10 µM, and varied substrate concentration as indicated. The derived kinetic parameters were summarized in Fig. 1i.

Figure S3

a

Sample		dC	dG	dT	dA	d6mA	dmA/(dA+d6mA)
DNA alone	1	5.601	4.000	3.052	0.969	0.000	0
	2	5.656	4.000	3.056	0.978	0.000	0
Sinefungin	3	5.783	4.000	3.120	1.008	0.005	0.52 %
	4	5.819	4.000	3.132	1.014	0.005	0.51 %
SAM	5	5.740	4.000	3.087	0.053	1.019	95.03 %
	6	5.801	4.000	3.124	0.054	1.029	95.06 %

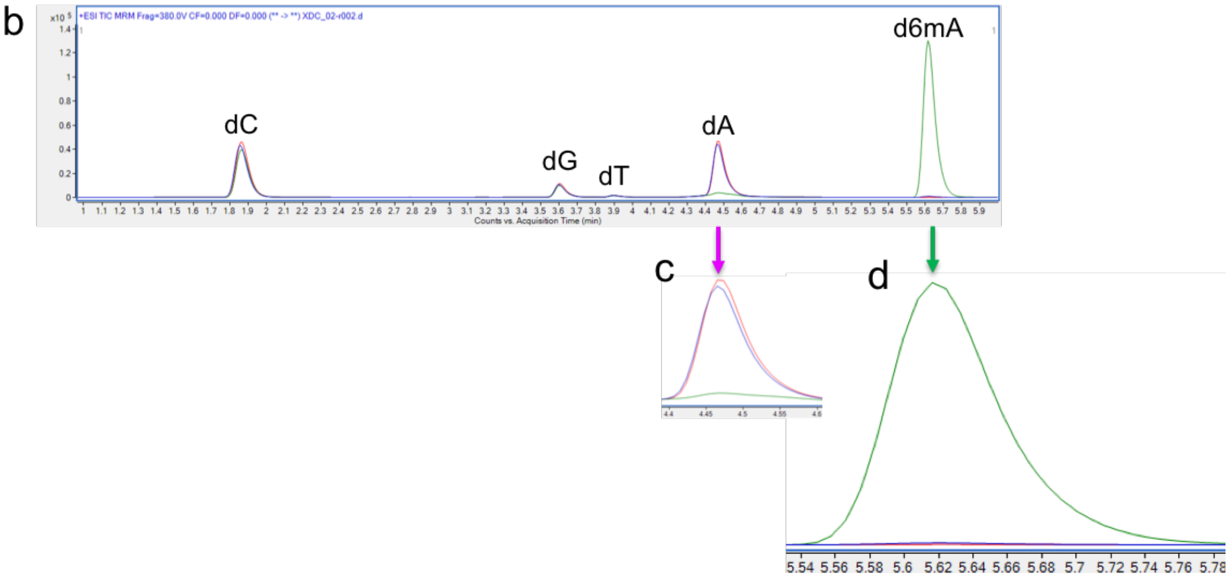

Fig. S3. **a**, Summary of quantitative MS measurement, normalized to dG amount (N=2). We observed residual N6mA (~0.5%) in the presence of Sinefungin, probably due to the co-purification of SAM with the enzyme complex. **b**, Superimposition of the total ion chromatograms of all monitored nucleosides from three samples (green: DNA + MettL3-14 + SAM; blue: DNA + MettL3-14 + Sinefungin; red: DNA alone). **c** and **d**, Enlarged multiple reaction monitoring chromatogram for dA (panel **c**) and d6mA (panel **d**).

Figure S4

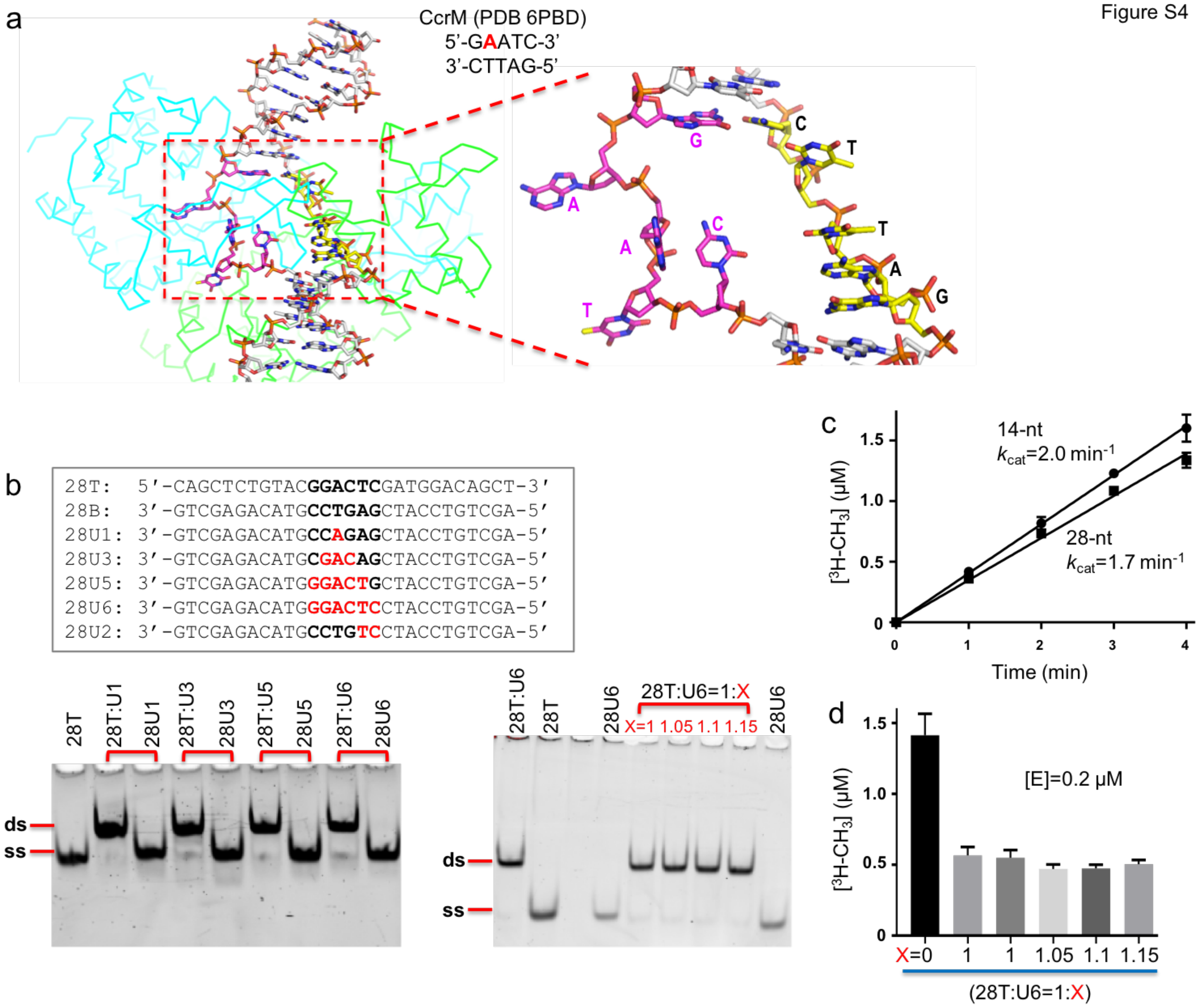

Fig. S4. a, CcrM opens a bubble at its DNA recognition site (PDB 6PBD). **b**, Oligonucleotides (28-mer) were annealed to form canonical double helix (28T:B), mismatched pairs involving target adenine (28T:U1, 28T:U3, 28T:U5, and 28T:U6), and mismatches pairs outside of adenine (28T:U2). **c**, MettL3-14 has approximately same activities on short (14-nt) and long (28-nt) ssDNA. **d**, Excess of bottom strand (28T:U6) does not affect activity on dsDNA.

Protein expression and purification

Human MettL3-MettL14. The original MettL3 and MettL14 clones were kindly provided by Dr. Chuan He (University of Chicago). DNA fragments encoding the MettL3 (residues 1-580) and MettL14 (residues 1-456) amplified separately by PCR and sub-cloned into pBacMam2-DiEx-LIC. The resulting plasmids were transformed into DH10Bac™ Competent E. coli (Invitrogen) and a recombinant viral DNA bacmids were purified and followed by a recombinant baculovirus generation in Sf9 insect cells. The Sf9 cells grown in HyQ® SFX insect serum-free medium (TermoScientific) and were co-infected with 10 ml of P3 viral stocks per 0.8L of suspension cell culture and incubated at 27°C using a platform shaker set at 100 RPM. The cells were collected after 72 h of post infection time, when viability dropped to 70-80%.

Harvested cells were re-suspended in PBS, 1X protease inhibitor cocktail (100 X protease inhibitor stock in 70% ethanol (0.25 mg/ml Aprotinin, 0.25 mg/ml Leupeptin, 0.25 mg/ml Pepstatin A and 0.25 mg/ml E-64) and 2X Roche complete EDTA-free protease inhibitor cocktail tablet. The cells were lysed chemically by rotating 30 min with NP40 (final concentration of 0.6%), 50 U/mL benzonase nuclease (Sigma), 2 mM 2-mercaptoethanol and 10% glycerol, followed by sonication at frequency of 7 (10" on/10" off) for 2 min (Sonicator 3000, Misoni). The crude extract was clarified by high-speed centrifugation (60 min at 36,000 ×g at 4˚C) by Beckman Coulter centrifuge.

The recombinant protein was purified by incubating the cleared lysate with equilibrate Anti-FLAG M2 Affinity agarose gel (Sigma, Cat # A2220) for 3 h rotating, followed by wash with 10 CV TBS (50 mM Tris-HCl, with 150 mM NaCl, pH 7.4) with 2 mM 2-mercaptoethanol, 1X protease inhibitor cocktail [100X protease inhibitor stock in 70% ethanol (0.25 mg/ml Aprotinin, 0.25 mg/ml Leupeptin, 0.25 mg/ml Pepstatin A and 0.25 mg/ml E-64)] and 1X Roche

complete EDTA-free protease inhibitor cocktail tablet. The recombinant protein was eluted by competitive elution with a solution containing 100 μg/mL FLAG peptide (Sigma, Cat # F4799) in TBS in addition of 1 mM dithiothreitol and 10% glycerol. The purity of the complex was judged by sodium dodecyl sulfate polyacrylamide gel electrophoresis (SDS-PAGE). The protein complexes were concentrated and flash frozen.

Assays of methylation reactions

Using tritiated SAM by scintillation. Reactions tracking product formation by the incorporation of tritium (^{3}H) were conducted in the reaction buffer 50 mM HEPES pH 7.5, 5 mM NaCl, 1 mM DTT, and 20 μM SAM using a ratio of 1:10 for labeled [methyl-^{3}H]SAM to unlabeled SAM. Prior to a reaction the 1:10 ratio of 50 μM SAM stock was prepared by using 1 mM cold SAM (supplied in the Promega MTase-Glo Kit) and a 59 μM stock of [methyl-^{3}H]SAM (1 μCi [16.9 Ci/mmol], PerkinElmer Lot# 2532638). DNA and RNA oligonucleotides were purchased from IDT or Sigma.

The reactions for each enzyme were conducted under the same steady state conditions with [S]=10 μM oligos, [SAM]=20 μM, and [E]=2 μM or 0.2 μM. Reactions were initiated by the addition of substrate into a reaction mixture of enzyme and SAM at 37°C, 5 μL aliquots of reaction sample were quenched and blotted onto Amersham Hybond-N$^+$ membrane and immediately submerged into 300 mL of 50 mM monophosphate buffer. After collecting all points for a reaction set (N=3), the Amersham samples were washed for three consecutive 5 min cycles at 90 rpm on an orbital bench top shaker followed by two 5 min washes with 200 proof ethanol after which they were air dried for 20 minutes. The samples were then placed into 20 mL glass scintillation vials with 10 mL of RPI BioSafe 2 scintillation cocktail and counted using a

PerkinElmer Tri-Carb 3110 TR in DPM mode with a 3 min integration time. Background specific to each wash was subtracted from all data points, and the data was plotted using GraphPad Prism 7.03.

The initial velocity reactions for K_m determination were conducted under steady state conditions with [SAM]=10 μM and [E]= 0.2 μM. Reactions (2 min at 37°C) were initiated by the addition of substrate (ssDNA or ssRNA) into a reaction mixture of enzyme and SAM in 50 mM Hepes, pH 7.5, 1 mM MnCl, 3 mM NaCl, and 1 mM DTT. The kinetic data was collected with N=3 and fitted with the Michaelis-Menten equation. The standard deviation associated with the second order substrate specificity constant, k_{cat}/K_m, was calculated using an error of propagation equation.

Promega bioluminescence assay (MTase-GloTM). Reactions were carried out in duplicates with 20 μl reaction mixture containing [S]=10 μM oligos, [SAM]=20 μM, [E]=2 μM or 0.2 μM in 20 mM Tris-HCl (pH 8.0), 50 mM NaCl, 0.1 mg/ml BSA and 1 mM DTT at room temperature (~22 °C) overnight (16 h) (Fig. 1d) or for 30 min (Fig. 1e). Reactions were terminated by addition of TFA to a final concentration of 0.1% (v/v) and a 5-μl mixture was transferred to a low-volume 384-well plate. The activity was measured using a bioluminescence assay (Promega MTase-Glo) in which the reaction by-product SAH is converted into ATP in a two-step reaction and ATP can be detected through a luciferase reaction (Ref. S24). The luminescence signal was measured by a Synergy 4 Multi-Mode Microplate Reader (BioTek).

Reaction products visualized by autoradiography or fluorography. Reactions visualized by film were conducted in the reaction buffer (50 mM HEPES pH 7.5, 5 mM NaCl, 1 mM DTT, and

20 μM SAM) using pure tritium labeled [methyl-^{3}H]SAM without additional unlabeled SAM. The reactions were conducted at room temperature (~22 °C) and allowed to progress overnight, after which reactions were blotted directly onto an Amersham Hybond-N$^+$ membrane followed by three 5 min wash steps in 50 mM monophosphate buffer and air dried.

Amersham Hyperfilm ECL was exposed to the spotted membranes for 16 h at -80°C. The washing technique described reduces the background DPM to below 1 x 10^5 counts for an area equivalent to the area occupied by the sample on the membrane, while samples of reactions had DPM in excess of 1 x 10^6 counts as determined by scintillation counting. After the film for autoradiography was developed the membrane was processed with PerkinElmer EN^{3}HANCE solution and allowed to air dry, followed by a 16 h exposure at -80°C with the Amersham Hyperfilm ECL.

ChIPseq data analysis. Raw fastq data (GSE92257 and GSE94613) were downloaded from NCBI Gene Expression Omnibus (GEO) (https://www.ncbi.nlm.nih.gov/geo/). The raw data were mapped to mouse mm10 version or human hg19 version reference genome using Bowtie2 (Ref. S25). The uniquely mapped reads were used for downstream peak calling analysis. MACS2 (Ref. S26) was used to call peaks against input with default parameters. The DNA sequences within the Mettl3 and Mettl14 peaks located around transcription start sites (± 2kb) were extracted with RRACH motif to perform motif analysis. MEME (http://meme-suite.org/tools/meme) was used to plot the motif logos with default parameters. UCSC genome browser was used to visualize ChIP-seq signal enrichment.

Substrate specificity analysis using triple-quadrupole mass spectrometry. To prepare the

samples for the MS measurement, each reaction consisted of 20 μM of oligo 6T (5'-CCGCGG**A**CTCTGCT-3') as substrates with or without 2.0 μM of MettL3-14 complex, in the presence of 40 μM SAM or Sinefungin, for 1 h at room temperature (~22 °C). The DNA alone sample was used as a control. The reaction samples were digested for 1 h at 37 °C to nucleosides using Nucleoside Digestion Mix (New England Biolabs). LC-MS/MS analysis was performed in duplicate by injecting digested samples on an Agilent 1290 UHPLC equipped with a G4212A diode array detector and a 6490A triple quadrupole mass detector operating in the positive electrospray ionization mode (+ESI). UHPLC was carried out on a Waters XSelect HSS T3 XP column (2.1 × 100 mm, 2.5 μm) with the gradient mobile phase consisting of methanol and 10 mM aqueous ammonium formate (pH 4.4). MS data acquisition was performed in the dynamic multiple reaction monitoring (DMRM) mode. Each nucleoside was identified in the extracted chromatogram associated with its specific MS/MS transition: dC $[M+H]^+$ at m/z 228→112, dG $[M+H]^+$ at m/z 268→152, dT $[M+Na]^+$ at m/z 265→149, dA $[M+H]^+$ at m/z 252→136, d5mC $[M+H]^+$ at m/z 242→126, and d6mA $[M+H]^+$ at m/z 266→150. External calibration curves with known amounts of the nucleosides were used to calculate their ratios within the samples analyzed and all results are normalized to dG amount.

Supplementary References

S1 Pingoud, A., Wilson, G. G. & Wende, W. Type II restriction endonucleases--a historical perspective and more. *Nucleic Acids Res* **42**, 7489-7527 (2014).

S2 Messer, W. & Noyer-Weidner, M. Timing and targeting: the biological functions of Dam methylation in E. coli. *Cell* **54**, 735-737 (1988).

S3 Berdis, A. J. *et al.* A cell cycle-regulated adenine DNA methyltransferase from Caulobacter crescentus processively methylates GANTC sites on hemimethylated DNA. *Proc Natl Acad Sci U S A* **95**, 2874-2879 (1998).

S4 Bestor, T. H. The DNA methyltransferases of mammals. *Hum Mol Genet* **9**, 2395-2402 (2000).

S4.1 Lentini A. *et al*. A reassessment of DNA-immunoprecipitation-based genomic profiling. *Nature Methods* **15**, 499-504 (2018).

S4.2 Schiffers, S. et al. Quantitative LC-MS Provides No Evidence for m^6 dA or m^4 dC in the Genome of Mouse Embryonic Stem Cells and Tissues. *Angew Chem Int Ed Engl* **56**, 11268-11271 (2017).

S5 Xiao, C. L. *et al.* N(6)-Methyladenine DNA Modification in the Human Genome. *Mol Cell* **71**, 306-318 (2018).

S6 Woodcock, C. B., Yu, D., Zhang, X. & Cheng, X. Human HemK2/KMT9/N6AMT1 is an active protein methyltransferase, but does not act on DNA in vitro, in the presence of Trm112. *Cell Discovery* **5**, 50 (2019).

S7 Li, W., Shi, Y., Zhang, T., Ye, J. & Ding, J. Structural insight into human N6amt1–Trm112 complex functioning as a protein methyltransferase. *Cell Discovery* **5**, 51 (2019).

S8 Knuckles, P. *et al.* RNA fate determination through cotranscriptional adenosine methylation and microprocessor binding. *Nat Struct Mol Biol* **24**, 561-569 (2017).

S9 Haussmann, I. U. *et al.* m(6)A potentiates Sxl alternative pre-mRNA splicing for robust Drosophila sex determination. *Nature* **540**, 301-304 (2016).

S10 Fedeles, B. I., Singh, V., Delaney, J. C., Li, D. & Essigmann, J. M. The AlkB Family of Fe(II)/alpha-Ketoglutarate-dependent Dioxygenases: Repairing Nucleic Acid Alkylation Damage and Beyond. *J Biol Chem* **290**, 20734-20742 (2015).

S11 Yang, B., Li, X., Lei, L. & Chen, J. APOBEC: From mutator to editor. *J Genet Genomics* **44**, 423-437 (2017).

S12 Ko, M. *et al.* Modulation of TET2 expression and 5-methylcytosine oxidation by the CXXC domain protein IDAX. *Nature* **497**, 122-126 (2013).

S13 Shen, Q. *et al.* Tet2 promotes pathogen infection-induced myelopoiesis through mRNA oxidation. *Nature* **554**, 123-127 (2018).

S14 Guallar, D. *et al.* RNA-dependent chromatin targeting of TET2 for endogenous retrovirus control in pluripotent stem cells. *Nat Genet* **50**, 443-451 (2018).

S15 Bujnicki, J. M., Feder, M., Radlinska, M. & Blumenthal, R. M. Structure prediction and phylogenetic analysis of a functionally diverse family of proteins homologous to the MT-A70 subunit of the human mRNA:m(6)A methyltransferase. *Journal of molecular evolution* **55**, 431-444 (2002).

S16 Woodcock, C. B., Yakubov, A. B. & Reich, N. O. Caulobacter crescentus Cell Cycle-Regulated DNA Methyltransferase Uses a Novel Mechanism for Substrate Recognition. *Biochemistry* **56**, 3913-3922 (2017).

S17 Ougland, R. *et al.* AlkB restores the biological function of mRNA and tRNA inactivated by chemical methylation. *Mol Cell* **16**, 107-116 (2004).

S18 He, Y. *et al.* Near-atomic resolution visualization of human transcription promoter opening. *Nature* **533**, 359-365 (2016).

S19 Reich, N. O., Dang, E., Kurnik, M., Pathuri, S. & Woodcock, C. B. The highly specific, cell cycle-regulated methyltransferase from Caulobacter crescentus relies on a novel DNA recognition mechanism. *J Biol Chem* **293**, 19038-19046 (2018).

S20 Ping, X. L. *et al.* Mammalian WTAP is a regulatory subunit of the RNA N6-methyladenosine methyltransferase. *Cell Res* **24**, 177-189 (2014).

S21 Little, N. A., Hastie, N. D. & Davies, R. C. Identification of WTAP, a novel Wilms' tumour 1-associating protein. *Hum Mol Genet* **9**, 2231-2239 (2000).

S22.1 Schwartz, S. *et al*. Perturbation of m6A writers reveals two distinct classes of mRNA methylation at internal and 5' sites. *Cell Rep* **8**, 284-296 (2014).

S21.2 Knuckles, P. *et al*. Zc3h13/Flacc is required for adenosine methylation by bridging the mRNA-binding factor Rbm15/Spenito to the m^6A machinery component Wtap/Fl(2)d. *Genes Dev* **32**, 415-429 (2018).

S21.3 Wen J. *et al*. Zc3h13 Regulates Nuclear RNA m^6A Methylation and Mouse Embryonic Stem Cell Self-Renewal. *Mol Cell* **69**, 1028-1038 (2018).

S22 Wang, W. *et al.* Epigenetic DNA Modification N(6)-Methyladenine Causes Site-Specific RNA Polymerase II Transcriptional Pausing. *J Am Chem Soc* **139**, 14436-14442 (2017).

S23 Hsiao, K., Zegzouti, H. & Goueli, S. A. Methyltransferase-Glo: a universal, bioluminescent and homogenous assay for monitoring all classes of methyltransferases. *Epigenomics* **8**, 321-339 (2016).

S24 Langmead, B. & Salzberg, S. L. Fast gapped-read alignment with Bowtie 2. *Nat Methods* **9**, 357-359 (2012).

S25 Zhang, Y. *et al.* Model-based analysis of ChIP-Seq (MACS). *Genome Biol* **9**, R137 (2008).
